# Evaluation of expression and serum concentration of anti-Mullerian hormone as a follicle growth marker following consumption of fennel and flaxseed extract in first-generation mice pups

**DOI:** 10.1186/s12906-021-03267-5

**Published:** 2021-03-12

**Authors:** Fahimeh Pourjafari, Tahereh Haghpanah, Fariba Sharififar, Seyed Noreddin Nematollahi-Mahani, Ali Afgar, Massood Ezzatabadipour

**Affiliations:** 1 Anatomical Sciences Department, School of Medicine, Kerman University of Medical Sciences, P.O. Box:76169-14115, Kerman, Iran; 2 Herbal and Traditional Medicines Research Center, Department of Pharmacognosy, Faculty of Pharmacy, Kerman University of Medical Sciences, Kerman, Iran; 3 Research Center for Hydatid Disease in Iran, Kerman University of Medical Sciences, Kerman, Iran

**Keywords:** Anti-Müllerian hormone, Fennel, Flaxseed, Ovarian follicular growth, First-generation mice pups

## Abstract

**Background:**

The aim of the present study was to assess the expression and serum level of AMH in first-generation female mice pups following fennel and flaxseed consumption.

**Methods:**

Twenty pregnant NMRI mice were allocated into four groups including control (CTL), fennel (FV), flaxseed (LU) and FV+ LU. Sixty-four female offsprings after lactation period, received the same regimen as their mothers for 56 and 240 days. The ovarian follicles development, serum concentration of AMH, as well as gene and protein expression of AMH were evaluated in the female offsprings at post-natal day 56 (PND56) and 240 (PND240).

**Results:**

The number of total growing follicles were raised in the FV group in compression to the all experimental groups. In contrast, LU group showed a marked decrease in their numbers. The highest level of serum AMH was seen in the FV-diet mice, whereas LU negatively affected it. The expression level of AMH also increased in the FV and FV + LU groups, while a reduction was observed in the LU group. As well, IHC data showed that the number of AMH-positive cells in almost ovarian follicles of FV and FV + LU-treated mice was in compared to those of the LU group.

**Conclusions:**

The overall effect of fennel treatment (alone and in combination with flaxseed) on ovary might be maintain primordial follicle storage through increased expression and serum level of AMH.

## Background

The exiting of the follicles from the quiescent pool and their entry into the growing follicle cohort are accompanied by the proliferation of granulosa cells and increased serum levels of anti-Mullerian hormone (AMH). In other words, there is a direct relationship among primordial follicle pool, growing follicles and serum levels of AMH [1]. Hence, serum AMH level measurement is known as a follicle growth marker and an assessment test for ovarian aging [1]. Thus, the initial size of the primordial follicle pool is a major determinant of reproductive lifespan [2]. As a member of the transforming growth factor-beta (TGF-β) family, AMH is produced postnatally by granulosa cells of primary follicles until large antral follicles develop in the ovarian tissue [3].

The serum levels of AMH increase slowly until puberty, gradually decline in adulthood and reach the lowest levels in menopause [3]. While AMH produced by the growing follicles locally inhibits primordial to primary follicle conversion, resulting in the maintenance of primordial follicle storage [3], it has stimulatory effects on the growth of preantral follicles in primates [4]. Therefore, it can be expected that higher serum levels of AMH lead to the exiting of fewer follicles (primordial) from the ovarian pool. The depletion of ovarian follicles is an age-dependent trend that begins from the middle of embryonic life, and it is expected to complete at the 74th year of life [5]. When the number of follicles reaches about 1000, menopause begins [6], which, in addition to the ending of women^’^s reproductive life, is the beginning of unpleasant changes in the woman^’^s physical and mental life [7]. All of these events are due to severe hormonal changes [7].

The improving potential of several herbs on ovarian function has been reported in various studies. Among different plants, two medicinal herbs, fennel, and flaxseed are reported to be effective in maintaining the ovarian follicular reserve (OFR) due to their antioxidant and phytoestrogenic properties [8–11].

Fennel (*Foeniculum vulgare,* FV), one of the oldest medicinal herbs, shows a wide range of clinical functions, including anti-inflammatory and anti-cancer activities, as well as estrogenic effects. The antioxidant effect of the ethanolic extract of fennel on female mice has also been confirmed [12, 13]. This biennial plant could inhibit free radicals due to the high content of antioxidant compounds such as polyphenols and flavonoids [10]. Our recent study indicated that fennel extract and seed administration (500 and 1000 mg/kg/BW) in pregnancy and lactation periods could improve F1 folliculogenesis, fennel extract at a dose of 500 mg/kg showed a more pronounced effect [14]. Another research also showed that alcoholic extract of fennel (100^_^200 mg/kg) could improve folliculogenesis due to its estrogenic property [15]. Moreover, fennel extract alone and in combination with flaxseed could improve offspring OFR at PND1, PND56 and PND240 because of antioxidant and anti-apoptotic properties [11]. The protective effect of fennel on the ovaries of cyclophosphamide-treated mice was also demonstrated by Hassanpour et al. [9].

Flaxseed (*Linum usitatissimum,* LU) is a rich source of lignans with a wide range of biological activity including antioxidant, antitumor, and weak estrogenic and anti-estrogenic effects [16, 17]. The strengthening of the body’s antioxidant capacity using flaxseed is attributed to its high cysteine and methionine content [18]. In females, its dose and duration of intake have relatively different effects on the reproductive system. Treatment of animals with 300 mg/kg body weight of LU for 25 days increased body, ovary, and uterus weight and led to precocious puberty, whereas at higher doses, it induced negative feedback [19].

Keeping in mind that serum AMH assay is an approved method for predicting primordial follicle pool size and ovarian aging in mice and women [20] and the complicated effects of FV and LU on the adult female reproductive system, the current study was aimed to investigate the effect of hydroalcoholic extracts of FV, LU, and their combination on the AMH expression at the cellular, molecular and hormonal levels as a follicle growth marker in the ovarian tissue of first-generation female mice pups.

## Methods

### Fennel and flaxseed extract preparation

Fennel (Voucher number: KF1466) and flaxseed (Voucher number: KF1628) were acquired from the Herbarium Center at the Department of Pharmacognosy, Kerman University of Medical Sciences, Kerman, Iran (KMU) and authenticated by the Department of Pharmacognosy Faculty of Pharmacy. The extracts of fennel and flaxseed were prepared by the warm maceration method. Briefly, dried seeds were milled and passed through a sieve (mesh 300). About 100 g of powdered seeds were soaked in 80% ethanol for 72 h. After that, the extracts were pooled and concentrated under vacuum conditions, dried in an oven at 40 °C for 48 h, and stored at − 20 °C for subsequent experiments [21].

### Animals and study design

All animals in the research were purchased from the animal house affiliated with Afzalipour School of Medicine, Kerman, Iran. They were housed in a 12-h light-dark cycle starting from 7:00 am to 7:00 pm at 21 ± 2 °C room temperature with ad libitum access to rodent chow and water, and treated in compliance with the guidelines for the care and use of animals approved by the Institutional Ethics Committee of Kerman University of Medical Sciences, Kerman, Iran (approval number IR.KMU.REC.1397.148).

**Fig. 1:**
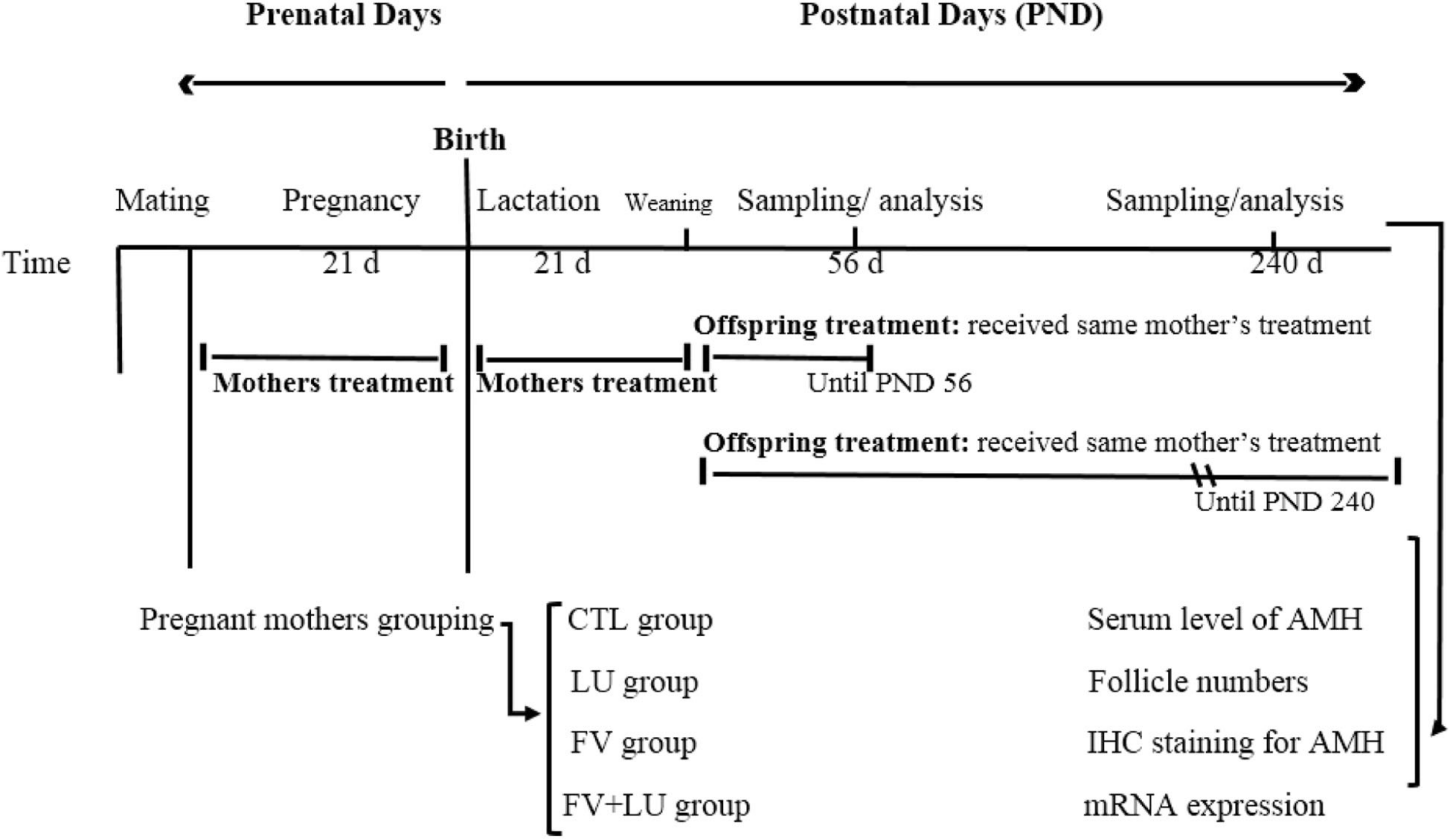
Schematic representation of the feeding protocol of different groups of mothers and offspring. CTL group: control group; LU group: the animals received 500 mg/kg/day hydroalcoholic extract of flaxseed; FV group: the animals received 500 mg/kg/day hydroalcoholic extract of fennel; FV + LU group: the animals received hydroalcoholic extract of flaxseed and fennel together (500 mg/kg/day). LU: *Linum Usitatissimum*. FV: *Foeniculum vulgare*, FV + LU: *Foeniculum vulgare*+ *Linum usitatissimum*. AMH: anti Mullerian hormone, IHC: immunohistochemistry

As in the experimental design in Fig. 1, thirty NMRI (Naval Medical Research Institute) mice, twenty females and ten males, 8–10 weeks old and weighing 25–30 g with proven fertility, were used for mating. The animals were housed in cages containing wood fiber bedding (3 mice per cage). In each cage, two adult female mice were randomly coupled with one male mouse at 8 pm. The next morning, female mice with a vaginal plaque were considered pregnant (pregnancy day 0, PD0). Pregnant animals were randomly grouped into four groups (*n* = 5): control group (CTL): the animals were fed with regular rodent food, FV group: the animals received 500 mg/kg/day hydroalcoholic extract of fennel [22], LU group: the animals received 500 mg/kg/day hydroalcoholic extract of flaxseed [23], and FV + LU group: the animals received a hydroalcoholic extract of fennel and flaxseed together (500 mg/kg/day). The extracts were mixed with the animals’ diet. Treatment of the pregnant mothers started at PD1 and continued during pregnancy and lactation period.

Twenty pregnant mothers were randomly divided into four pentad groups (control, fennel, flaxseed, and fennel plus flaxseed). Sixteen female pups of five mothers in each of the four groups were randomly assigned to be evaluated on days 56 and 240 so that a maximum of two pups per mother were in the same group. Eight octet groups were eventually formed (a total of 64 first-generation offspring). Pups received the same treatment as mothers after the end of lactation (PND21) until day 56 and day 240 (Fig. 1).

### Determination of serum level of AMH

The female offspring were anesthetized by ketamine (5-10 mg/kg) and xylazine (5 mg/kg) (the mixture of ketamine as a dissociative agent and xylazine as a 2-adrenoreceptor agonist is one of the well-established general anesthetic modalities commonly used for laboratory mice) on PND56 and PND240 and blood were aspirated from the left ventricle, and centrifuged at 2000-2500 g for 10 min; the serum was collected and kept at - 20 °C for subsequent experiments. AMH was measured using appropriate laboratory ELISA kits (cat.no.CK-E90200, Hangzhou East biopharma, Ltd., China) for mice as recommended by the manufacturer [24]. After sacrificed by cervical dislocation, the right ovaries were collected rapidly and fixed in 10% buffered formaldehyde for histological analysis.

### Evaluation of follicle numbers and stages in the ovary

On PND56 and PND240, the harvested ovaries of female offspring were fixed by immersion in 10% buffered formaldehyde at room temperature for 48 h, dehydrated in decreasing concentrations of ethanol (100, 95, 80, 70, and 50%) and embedded in paraffin. One slide was selected from every ten 5 μm-thick serial sections (8 sections from each ovary, *n* = 8). These sections were stained with hematoxylin and eosin (Merck, Darmstadt, Germany) (H&E) and evaluated under a light microscope (Olympus IX51, Tokyo, Japan) to determine follicle numbers and stages as follows:

1. Primary follicle: an oocyte surrounded by one layer of cuboidal granulosa cells.
2. Secondary follicle: two or three layers of cuboidal granulosa cells without any antral space.
3. Pre-antral follicle: more than four layers of granulosa cells with one or more independent antral spaces
4. Antral follicle: multiple layers of cuboidal granulosa cells with a defined large antrum [9].

### AMH assay through immunohistochemistry

Immunohistochemistry (IHC) staining of the 19th five-μm-thick paraffin-embedded section of the right ovary was performed to assess the ovarian expression of AMH protein as a follicle growth marker [25]. This section number was similar in all of the studied ovaries. The sections were incubated at 60 °C for 1 h, deparaffinized in xylene for 30 min, and rehydrated in decreasing concentrations of ethanol (100, 95, 80, 70 and 50%). Antigen retrieval was performed in 0.01 M citrate buffer (pH 6.0) and high microwave irradiation for 25 min. Endogenous peroxidase activity was eliminated by incubation in 3% H2O2 at room temperature for 30 min. The slides were washed three times in PBS-tween 20 (0.05%) for 5 min, followed by incubation in 0.5% triton for 10 min. The slides were incubated overnight in mouse anti-AMH monoclonal antibody (cat.no. ab24542, Abcam, Cambridge, MA, USA, 1:40) at 4 °C. The sections were rinsed three times in PBS-Tween 20 and were then incubated in the secondary antibody (DB detection kit - rabbit/mouse dual, HRP/DAB, DB Biotech, Popradska, Slovak Republic) for 1 h. After washing with PBS-Tween 20, 3, 3-diaminobenzidine (DAB) was added for 15 min. For nucleus staining, hematoxylin was used for 1 min, and the sections were then mounted. Negative control slides were incubated with PBS instead of the primary antibody. In each section (three sections from each ovary, *n* = 8), preantral and antral follicles were classified according to the follicle size [26] (diameter = x mm) (x < 0.1, 0.1 ≤ x < 0.2, 0.2 ≤ x < 0.3 and x ≥ 0.3). The number of the AMH-positive granulosa cells (AMH+) was recorded and scored accordingly in different size follicles AMH+ = 1 (follicle score: weak), AMH+ = 2 (follicle score: moderate), AMH+ = 3 (follicle score: strong), and AMH+ = 4 and higher (follicle score: very strong) [27]. Total AMH+ granulosa cell number was calculated in each group and analyzed statistically.

### RNA extraction and quantitative PCR

The expression levels of AMH in the left ovary of offspring on PND56 and PND240 were measured by the quantitative real-time PCR technique. Total RNA extraction was performed by TRIZOL (1 ml) and cold chloroform (200 μl) in RNAase-free microtubes containing ovarian tissue; they were centrifuged at 12000 g for 20 min at 4 °C. The supernatant was removed, absolute ethanol (100%) was added, and they were then incubated at − 20 °C. After 24 h of incubation, the solution was centrifuged at 1200 g for 20 min at 4 °C. The supernatant was removed, ethanol 70% was added, and it was centrifuged again at 1200 g for 20 min at 4 °C. The RNA pellet was dried at room temperature for 20 min. Finally, 50 μl RNAase-free water was added to each microtube and the absorbance of the sample was measured at 260 nm by NanoDrop and the 260/280 ratio was calculated. Complementary DNA (cDNA) was synthesized using the PrimeScript™ Reverse Transcriptase Kit (cat.no.YT4500, YTA, Tehran, IRAN) according to the manufacturer’s instructions. Triplicate qRT-PCR reactions consisted of 5 μl 2X qPCRSYBR Green master mix, 2 μl RNA-specific cDNA and 0.5 mM of each forward and reverse primers in a final volume of 10 μl were performed using the Rotor-Gene 6000 instrument (Corbett Research, Brisbane, Sydney, Australia). The reaction conditions were 15 min at 95 °C followed by 40 cycles of 95 °C for 15 s and 62.5 °C for 15 min. Beta actin was assigned as a housekeeping gene. The analysis was done by the comparative CT method (2^-ΔΔct^). The primers are shown in Table 1.

**Table 1:** Primers sequence for targeted genes

Gene	Primer sequence (5^**′**^-3^**′**^ orientation)	Annealing T. (°c)	GeneBank Accession no.
**AMH**	**Forward: TTG GTG CTA ACC GTG GAC TTC** **Reverse: CGG GAA TCA GAG CCA AAT AGA AA**	**62.5**	**NM_007445.2**
**β-actin**	**Forward: GTC CAC ACC CGC CAC CAG TT** **Reverse: GAG CCG TTG TCG ACG ACC AG**	**60**	**NM_0073930.5**

*T* temperature

### Statistical analysis

Data were analyzed by SPSS version 22 software. Data were tested for normality by using the One-sample Kolmogorov-Smirnov test. The parametric data was analyzed by the analysis of variance (ANOVA) followed by Tukey post-hoc and non-parametric data was analyzed by the Krus-kalwalis test. The values were presented as mean ± SEM. *p* ≤ 0.05, *p* < 0.01 and *p* < 0.001 were considered statistically significant.

## Results

### Serum level AMH determination

**Fig. 2:**
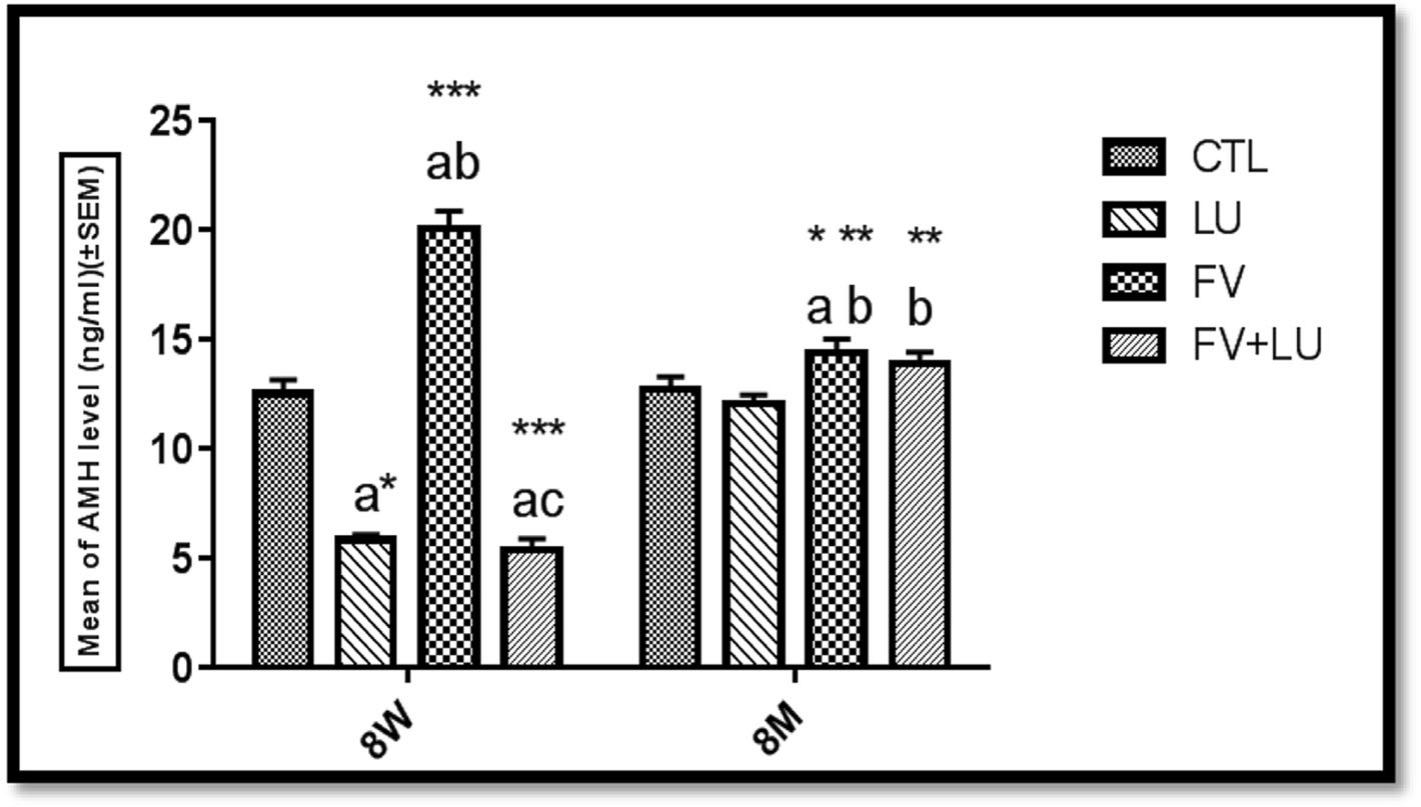
Serum level of AMH at PND56 (8w) and PND240 (8 m). Values were expressed as mean ± SEM, **a** significant difference versus the CTL; **b** significant difference versus the LU group; **c** significant difference versus the FV group. * *p* ≤ 0.05, ** *p* ≤ 0.01 and *** *p* ≤ 0.001. PND56: postnatal day 56, PND240: postnatal day 240, LU: (*Linum usitatissimum*)**.** FV: *Foeniculum vulgare*, FV + LU: *Foeniculum vulgare*+ *Linum usitatissimum*. 8w: 8 weeks, 8 m: 8 months

As shown in the Fig. 2, the serum level of AMH in the LU-treated animals (5.82 ± 0.26) was lower than that of the CTL group (12.5 ± 0.63) on PND56 (*p* = 0.001) whereas its level (12.05 ± 0.4) was close to that of the CTL group (12.71 ± 0.57) on PND240. Also, the AMH levels of the FV group on PND56 and PND240 were 20.06 ± 0.78 and 14.38 ± 0.64, respectively. Interestingly, these values were significantly higher than those of the CTL group (12.5 ± 0.63, 12.71 ± 0.57), and LU (5.82 ± 0.26, 12.05 ± 0.4) groups on PND56 (*p* = 0.001) and PND240 (*p* = 0.042, *p* = 0.01), respectively. Moreover, the AMH level in the FV + LU group was lower (5.36 ± 0.52, *p* = 0.001) compared to the CTL and FV groups on PND56 (*p* = 0.001); on PND240, a marked difference (12.06 ± 0.6, *p* = 0.019) was observed in comparison with the AMH level in the LU group (5.36 ± 0.52).

### Follicle numbers and stages

**Fig. 3:**
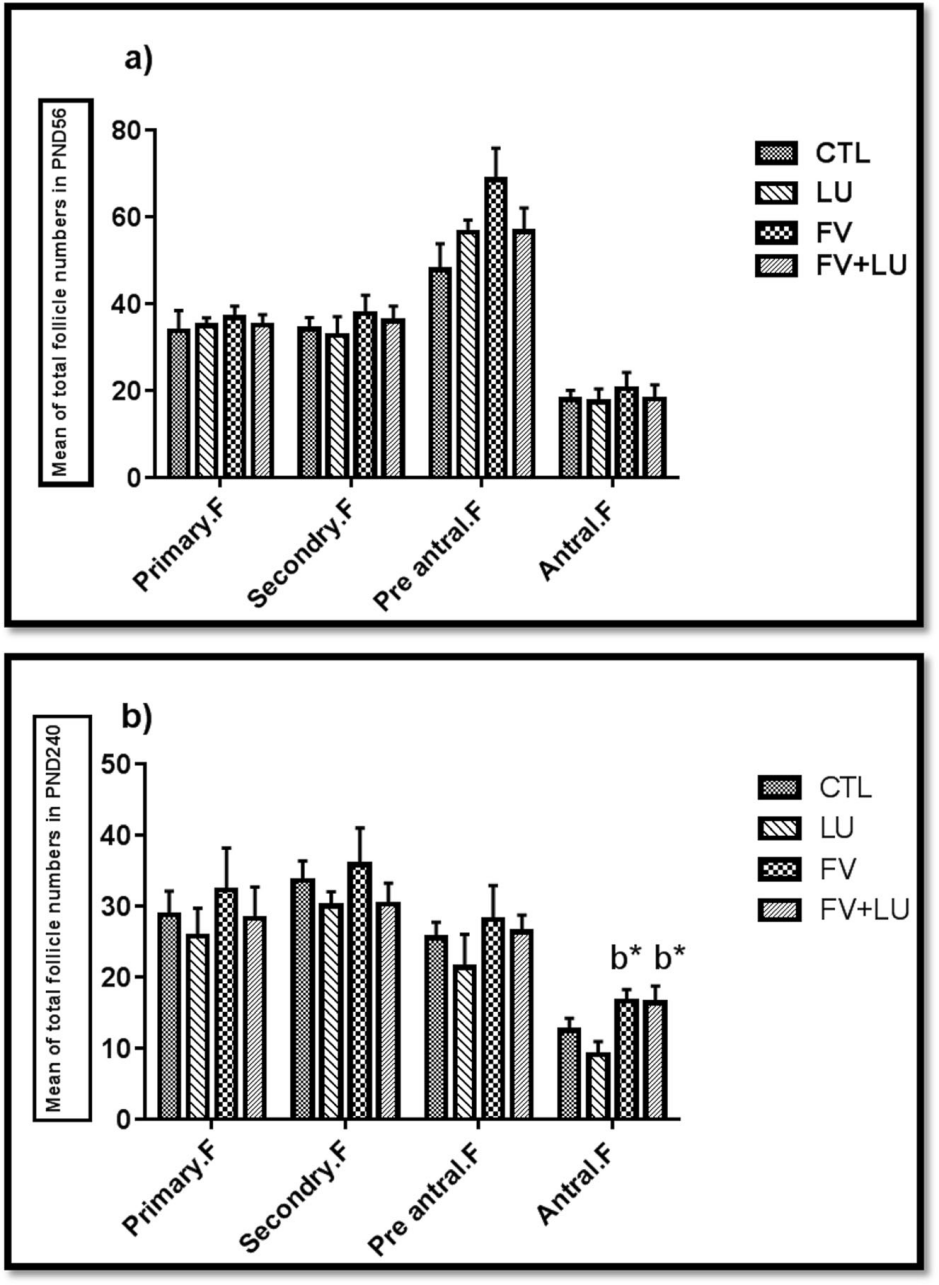
**a**, **b** Effects of fennel and flaxseed on the folliculogenesis in the mice ovaries at PND56 (**a**) and PND240 (**b**). The values were expressed as mean ± SEM, **b** Significant difference compared to the LU group, * *p* ≤ 0.05. PND56: postnatal day 56, PND240: postnatal day 240, LU: (*Linum usitatissimum*)**.** FV: *Foeniculum vulgare*, FV + LU: *Foeniculum vulgare*+ *Linum usitatissimum*

Results depicted in Fig. 2 showed that the number of primary, secondary, and pre-antral follicles on PND56 and PND240 was not significantly affected by any of the treatments. However, treatment of animals with fennel extract did have an improving effect. The number of antral follicles did not differ significantly among the groups on PND56 (Fig. 3a), whereas on PND240 (Fig. 3b), the number of antral follicles in the FV and FV + LU groups markedly increased compared to the LU group (*p* = 0.02 and *p* = 0.03, respectively).

### Immunohistochemistry detection of AMH

**Fig. 4:**
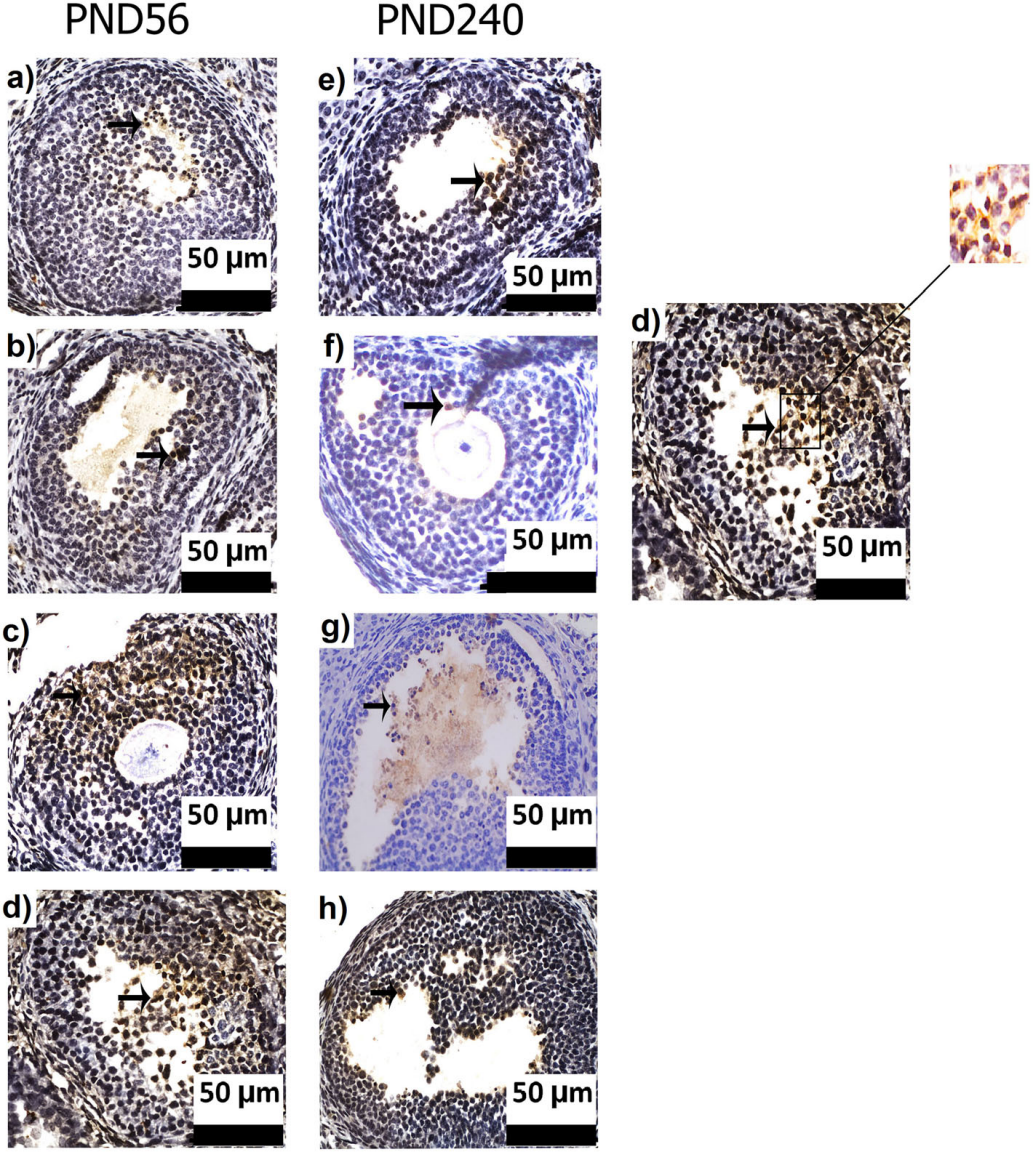
AMH immunostaining of antral follicles in different groups at PND56 and PND240 (left). AMH immunostaining of antral follicle with high magnification was illustrated; brown color in granulose cells (black arrow; →) shows positive AMH cell (2) that counterstained with hematoxylin. Scale bars is 50 μm. PND56: postnatal day 56, PND240: postnatal day 240, AMH: anti Mullerian hormone

The number of pre-antral and antral follicles with different diameters and the positive intensity of AMH were analyzed by the immunohistochemistry method. In the FV group, AMH-positive cells increased in all follicles irrespective of diameter, intensity or age. Analysis of immunohistochemistry data, based on weak, moderate, and strong intensity, showed no significant differences in the antral follicles of all diameters among experimental groups. However, only pre-antral follicles with moderate and strong intensities did not significantly differ in the experimental groups (Fig. 4, Tables 2 and 3).

**Table 2:** Mean number of pre-antral and antral follicles that positively stained for AMH with different sizes (X < 0.1, 0.1 ≤ X < 0.2, 0.2 ≤ X < 0.3, and X ≥ 0.3) and intensity (weak, moderate, strong, very strong) in F1 ovarian mice in PND56

Groups	Follicle diameter (mm)	PND56							
		Staining intensity of pre-antral follicle				Staining intensity of antral follicle			
		Weak	Moderate	Strong	Very strong	Weak	Moderate	Strong	Very strong
CTL	**X < 0.1**	0.12 ± 0.12	0	0	0	0.12 ± 0.12	0	0	0.12 ± 0.12
	**0.1 ≤ X < 0.2**	0.12 ± 0.12	0.12 ± 0.12	0.25 ± 0.16	0.25 ± 0.25	0	0.12 ± 0.12	0	0.12 ± 0.12
	**0.2 ≤ X < 0.3**	0.25 ± 0.16	0.12 ± 0.12	0.12 ± 0.12	0.37 ± 0.26	0.12 ± 0.12	0	0	0.12 ± 0.12
	**x ≥ 0.3**	0.25 ± 0.25	0.12 ± 0.12	0.12 ± 0.12	0.12 ± 0.12	0	0.12 ± 0.12	0.12 ± 0.12	0.12 ± 0.12
LU	**X < 0.1**	0	0	0	0	0	0	0	0.12 ± 0.12
	**0.1 ≤ X < 0.2**	0.25 ± 0.16	0	0.12 ± 0.12	0.12 ± 0.12	0	0	0.12 ± 0.12	0
	**0.2 ≤ X < 0.3**	0.25 ± 0.16	0.25 ± 0.16	0.12 ± 0.12	0.25 ± 0.16	0	0	0	0.12 ± 0.12
	**x ≥ 0.3**	0.37 ± 0.26	0.12 ± 0.12	0.12 ± 0.12	0.12 ± 0.12	0.12 ± 0.12	0	0	0.12 ± 0.12
FV	**X < 0.1**	0.25 ± 0.16	0.12 ± 0.12	0.12 ± 0.12	1.25 ± 0.45^ab**^	0.12 ± 0.12	0	0.12 ± 0.12	0.25 ± 0.16
	**0.1 ≤ X < 0.2**	0.37 ± 0.18	0.25 ± 0.16	0.37 ± 0.18	0.5 ± 0.26	0.12 ± 0.12	0.12 ± 0.12	0.12 ± 0.12	0.25 ± 0.16
	**0.2 ≤ X < 0.3**	0.37 ± 0.26	0.37 ± 0.18	0.25 ± 0.16	0.75 ± 0.31	0.12 ± 0.12	0	0	0.37 ± 0.18
	**x ≥ 0.3**	0.5 ± 0.18	0.25 ± 0.25	0.25 ± 0.16	0.12 ± 0.12	0.12 ± 0.12	0.12 ± 0.12	0.25 ± 0.16	0.5 ± 0.18
FV + LU	**X < 0.1**	0.37 ± 0.18	0	0	0.12 ± 0.12^c*^	0	0	0.12 ± 0.12	0
	**0.1 ≤ X < 0.2**	0.12 ± 0.12	0.12 ± 0.12	0.25 ± 0.16	0.62 ± 0.32	0.12 ± 0.12	0	0	0.12 ± 0.12
	**0.2 ≤ X < 0.3**	0.5 ± 0.37	0.37 ± 0.26	0.25 ± 0.25	0.62 ± 0.32	0.12 ± 0.12	0.12 ± 0.12	0	0.37 ± 0.18
	**x ≥ 0.3**	0^c*^	0	0.12 ± 0.12	0	0	0.12 ± 0.12	0.25 ± 0.16	0.25 ± 0.16

The values are expressed as (mean ± SEM). X means follicle diameter (mm). The values are comparable in the same column

*F1* first offspring, *PND56* postnatal day 56, *LU Linum usitatissimum***,** *FV Foeniculum vulgare*, *FV + LU Foeniculum vulgare*+ *Linum usitatissimum*

*, *p* < 0.05 and **, *p* < 0.01

^a^ significant difference versus the control group (CTL)

^b^ significant difference versus LU group

**Table 3:** Mean number of pre-antral and antral follicles that positively stained for AMH with different sizes (X < 0.1, 0.1 ≤ X < 0.2, 0.2 ≤ X < 0.3, X ≥ 0.3) and intensity (weak, moderate, strong, very strong) in F1 ovarian mice in PND240

Groups	Follicle diameter (mm)	PND240							
		Staining intensity of pre-antral follicles				Staining intensity of antral follicles			
		Weak	Moderate	Strong	Very strong	Weak	Moderate	Strong	Very strong
**CTL**	**X < 0.1**	0.87 ± 0.22	0.75 ± 0.36	0.25 ± 0.16	0.25 ± 0.16	0	0	0	0
	**0.1 ≤ X < 0.2**	0.12 ± 0.12	0.25 ± 0.16	0.25 ± 0.16	0.25 ± 0.16	0	0.25 ± 0.16	0.12 ± 0.12	1.87 ± 0.39
	**0.2 ≤ X < 0.3**	0	0.12 ± 0.12	0.12 ± 0.12	0	0	0	0.12 ± 0.12	0.12 ± 0.12
	**x ≥ 0.3**	0.12 ± 0.12	0	0.12 ± 0.12	0	0	0	0	0
LU	**X < 0.1**	0.37 ± 0.18	0.5 ± 0.26	0.25 ± 0.16	0.12 ± 0.12	0	0	0	0
	**0.1 ≤ X < 0.2**	0.25 ± 0.16	0.37 ± 0.18	0.12 ± 0.12	0.12 ± 0.12	0.12 ± 0.12	0.25 ± 0.16	0.25 ± 0.25	0.37 ± 0.18^a*^
	**0.2 ≤ X < 0.3**	0.5 ± 0.26	0.12 ± 0.12	0.25 ± 0.16	0	0	0	0	0
	**x ≥ 0.3**	0.12 ± 0.12	0	0	0	0	0.12 ± 0.12	0	0
FV	**X < 0.1**	0.5 ± 0.18	0.75 ± 0.25	0.12 ± 0.12	0.62 ± 0.26	0	0	0	0.25 ± 0.16
	**0.1 ≤ X < 0.2**	0.5 ± 0.26	0.37 ± 0.18	0.37 ± 0.18	1.125 ± 0.35^ab*^	0	0.12 ± 0.12	0.5 ± 0.26	2.37 ± 0.53^b**^
	**0.2 ≤ X < 0.3**	0.25 ± 0.25	0.12 ± 0.12	0.25 ± 0.16	0.37 ± 0.18	0.12 ± 0.12	0	0	0.5 ± 0.26
	**x ≥ 0.3**	0.25 ± 0.16	0	0.25 ± 0.16	0.12 ± 0.12	0	0.25 ± 0.25	0.12 ± 0.12	0.25 ± 0.25
FV + LU	**X < 0.1**	0.87 ± 0.35	0.37 ± 0.18	0.5 ± 0.18	0.62 ± 0.26	0	0.12 ± 0.12	0.12 ± 0.12	0.12 ± 0.12
	**0.1 ≤ X < 0.2**	0.12 ± 0.12	0.37 ± 0.18	0.5 ± 0.26	1.75 ± 0.25^b*^	0	0	0	1.5 ± 0.32^b*^
	**0.2 ≤ X < 0.3**	0.25 ± 0.25	0.12 ± 0.12	0.12 ± 0.12	0.12 ± 0.12	0	0.12 ± 0.12	0	0.12 ± 0.12
	**x ≥ 0.3**	0.12 ± 0.12	0	0.12 ± 0.12	0	0.12 ± 0.12	0.12 ± 0.12	0	0.12 ± 0.12

The values are expressed as (mean ± SEM). The values are comparable in the same column

*F1* first offspring, *PND240* postnatal day 240, *LU Linum usitatissimum*, *FV Foeniculum vulgare*, *FV + LU Foeniculum vulgare*+ *Linum usitatissimum*

*, *p* < 0.05 and **, *p* < 0.01

^a^ significant difference versus the control group (CTL)

^b^ significant difference versus LU group

As shown in Table 2, very strong intensity in pre-antral follicles, only in diameters less than 0.1 mm (x < 0.1), exhibited a marked increase in the FV group compared to the other experimental groups on PND56 (CTL: *p* = 0.003, LU: *p* = 0.003, and FV + LU: *p* = 0.013). Also, the mean number of weak-intensity pre-antral follicles with diameters ≥0.3 mm (x ≥ 0.3) in the FV group was significantly higher than that of the FV + LU group on PND56 (*p* = 0.25).

Furthermore, ovarian AMH immunohistochemistry of offspring aged 240 days (PND240) showed that the mean number of pre-antral (*p* = 0.013 and *p* = 0.042, respectively) and antral follicles (*p* = 0.003 and *p* = 0.014, respectively) with very strong intensity and diameter of 0.1 ≤ x < 0.2 markedly increased in the FV and FV + LU groups when compared to the LU group (Table 3).

Besides, on PND240, the mean number of follicles with very strong intensity and diameters of 0.1 ≤ x < 0.2 revealed that FV extract administration could lead to a significant increase in pre-antral follicles compared with the CTL group (*p* = 0.037). In contrast, based on the antral follicles, the LU group had a lower level of very strong antral follicles among different groups as a significant difference was observed in the number of follicles with diameters of 0.1 ≤ x < 0.2 when compared to the other groups (CTL: *p* = 0.01, FV: *p* = 0.003, FV + LU: *p* = 0.014) (Table 3).

### Evaluation of AMH expression with quantitative-RT-PCR technique

**Fig. 5:**
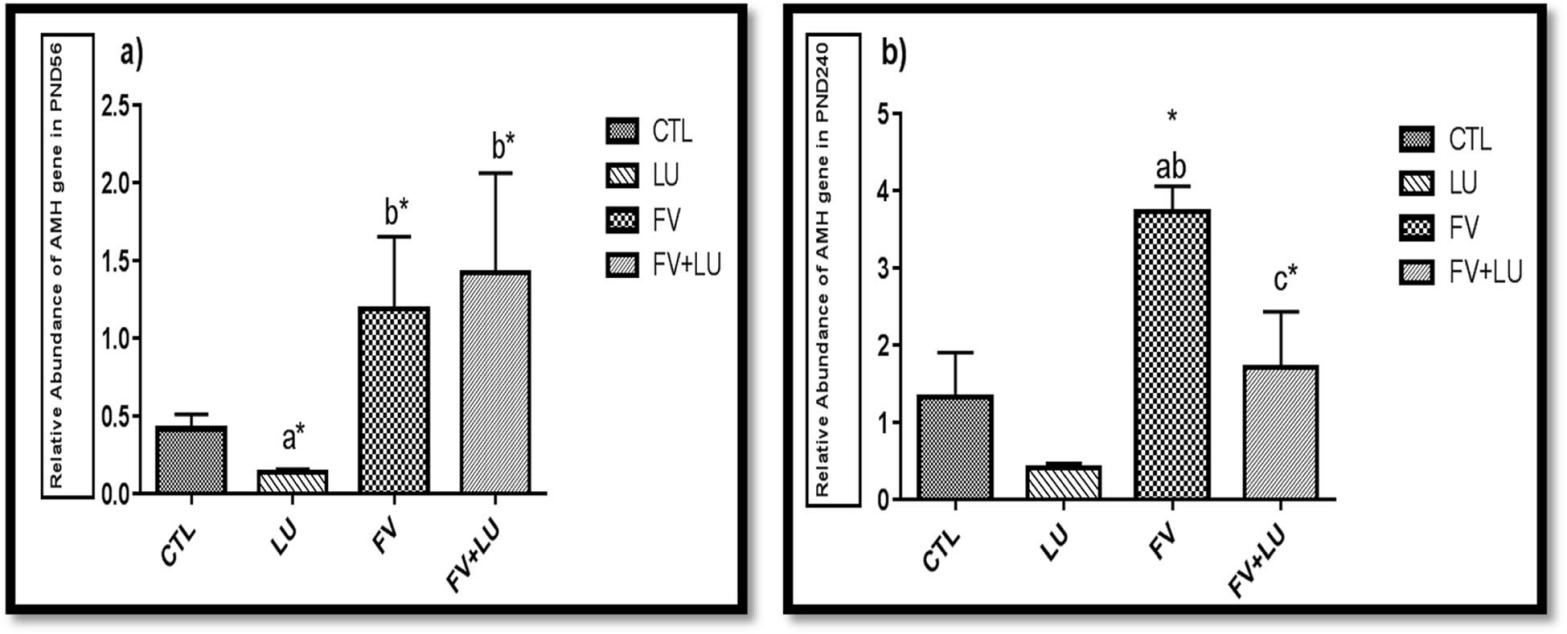
Expression of level of AMH in the left ovarian tissue of mice offspring after treatment with hydroalcoholic extract of fennel and flaxseed, alone and in combination. **a** significant difference versus CTL group; **b** significant difference versus LU group; **c** significant difference versus FV group; (*, *p* < 0.05). PND56: postnatal day 56, PND240: postnatal day 240, LU: (*Linum usitatissimum*)**.** FV: *Foeniculum vulgare*, FV + LU: *Foeniculum vulgare*+ *Linum usitatissimum*. AMH: anti Mullerian hormone

Quantitative RT-PCR analysis showed that AMH expression increased in the FV group compared to the other experimental groups on PND240 (*p* = 0.05) (Fig. 5). Also, daily intake of flaxseed extract alone markedly decreased the expression level of AMH compared to the CTL group on PND56 (*p* = 0.05) (Fig. 5). Expression of AMH in the FV or FV + LU groups increased significantly compared with the LU and CTL groups on PND56 (*p* = 0.05).

## Discussion

Considering that AMH is known as an inhibitor of new primordial follicle recruitment [28], an indicator of ovarian aging, and preserver of primordial follicle stock, the main objective of the present study was to evaluate the effect of two known medicinal herbs (fennel and flaxseed) on serum AMH and ovary expression in first-generation female mice pups. Briefly, our data indicated that fennel significantly increased serum and ovary expression level of AMH.

There is ample evidence for the biological effects of AMH on the ovary. The expression of 707 ovarian genes is under the influence of AMH [29]. Given the fact that granulosa cells postnatally produce AMH, primordial follicles do not express AMH. When squamous cells of the primordial follicle convert to cuboid cells, AMH expression begins [28, 30]. AMH expression begins and rises in growing follicles, which in addition to the stimulatory effect on the growth of pre-antral follicles in primates [4] prevents the recruitment of primordial follicles from the quiescent pool [3]. The 120-day travel of follicles from the reservoir to ovulation occurs in two stages: gonadotropin-independent (initial, early, paracrine control) and gonadotropin-dependent (cyclic, late, endocrine control), with initial recruitment being regulated by AMH and the cyclic phase by FSH [31]. The direct survey of primordial follicle storage and predicting female genital reproductive lifespan and ovary aging, chance of pregnancy and other clinical instances is not possible. Several methods such as ultrasonography, measurement of serum level of estradiol, inhibin B, FSH, and AMH are used, among which AMH is the most reliable [27] because serum level of estradiol, inhibin B, and FSH are gonadotrophin-dependent (under the influence of the hypothalamus-pituitary-gonadal axis) and their production occurs in the late stage [32, 33]. Developing follicles up to 4 mm (primary, secondary, pre-antral and antral follicles) show the highest expression of AMH [26]. Our data were in agreement with these findings. Although the number of growing follicles of the fennel-treated group was not significantly higher than that of the flaxseed-treated group, fennel treatment significantly increased the number of antral follicles (highest AMH-expressing follicles). It seems that FV promoted follicle growth because more antral follicles were observed on PND240 after both FV and FV + LU treatment. This change was associated with a significant increase in the serum level of AMH and ovarian tissue AMH expression in the fennel-treated group. Our data are consistent with evidence for AMH inducing pre-antral follicle growth to the antral, stage as discussed above, which provides valuable evidence on AMH stimulatory effects in mice. Also, fennel treatment brought AMH expression closer to the control group. Even though the ovarian expression of AMH in the control group on PND240 was higher than it was on PND56, the serum levels of AMH were also slightly higher on PND240 than they were on PND56; however, this difference was not significant due to unknown reasons. Because the method of assessing serum level and AMH expression was the same in all groups, the reason for the proximity of these values at the two ends of this period is unknown.

Primordial to primary follicle transition is regulated by stimulants PDGF (platelet-derived growth factor) [34], bFGF (basic fibroblast growth factor) [35], LIF (leukemia inhibitor factor) [36], KITL (kit ligand) [37], BMP4, BMP7 (bone morphogen protein) [38] and inhibitors AMH & SDF1 (stromal cell-derived factor 1) [30, 39] from three sources: oocyte (SDF1, PDGF), granulosa cells (AMH, LIF, KITL), and interstitial cells (BMP4, BMP7, FGF7). The dose-dependent modulating action of AMH in interplay with the stimulants and inhibitors of primordial follicles recruitment ultimately leads to the prevention of their entry into the growing follicles and retention of a greater number of primordial follicles in the ovarian reservoir. Another study showed that AMH-treated ovaries contained 40% fewer growing follicles [3] and AMH knockout mice had fewer primordial follicles [30]. While the onset of primordial follicle stock formation begins in the fetal period, it ends before birth in humans and immediately after birth in rodents such as mice, and some of them remain quiescent for years in humans and months in mice [40].

Regardless of the controversy surrounding the therapeutic benefit of medicinal herbs, they have attracted many people as an alternative treatment for controlling disease, because of increasing evidence of their effectiveness. The efficacy of medicinal plants can be attributed to a wide range of clinical properties, such as their anti-inflammatory, anti-cancer, and antioxidant effects [13, 41]. Therefore, the question of the present study was to evaluate the effect of fennel and flaxseed treatment during intrauterine life and lactation on serum levels and ovarian expression of AMH-gene and protein on post-natal day 56 (puberty onset) and 240 (menopause onset). To the best of our knowledge, no study has studied this so far. Our data indicated that fennel consumption alone and in combination with flaxseed could protect the ovarian primordial follicle storage through increasing the serum level of AMH and also tissue expression of AMH- gene and protein while consumption of flaxseed alone decreased these values. These findings are in line with our previous study, which confirmed the protective effects of fennel and flaxseed on OFR due to phenolic contents of these plants that have estrogenic and antioxidant activity. We also assessed the level of Rutin as flavonoid content in these extracts so that, high level of flavonoid content was observed in the fennel hydroalcoholic extract [11]. Probably the data of the current study are related to valuable content of flavonoids in the fennel extract. Although the anti-apoptotic effect of flaxseed on the granulosa cells has been reported earlier [42], our publishing data revealed that compared to flaxseed, fennel showed a more significant anti-apoptotic effect to protect ovarian follicles. As previously demonstrated, fennel treatment could protect ovarian follicles against the side effects of cyclophosphamide [9]. Fennel extract could induce folliculogenesis in mice and increase the number of growing follicles because of its estrogenic property [15]. The pharmacological effects of fennel on many organs have recently been highlighted in a review article [13]. Other research data showed the beneficial effect of fennel on kidney function [43]. Although our study results did not confirm the effectiveness of flaxseed on OFR compared to fennel, a report in 2018 indicated the ameliorative effect of flaxseed in polycystic rats  [44].

Based on our data and previous studies, an increase in the amount of growth hormone reflects, on the one hand, the volume of follicles secreting it, and on the other, the greater inhibition of the release of primordial follicles from the ovarian stock.

## Conclusion

Fennel treatment alone and in combination with flaxseed could promote follicle growth to antral stage, increase the serum AMH levels and ovarian tissue AMH expression, thus it seems that AMH has stimulatory effects in mice.

## Data Availability

The datasets used and/or analyzed during the current study are available from the corresponding author on reasonable request.

## Abbreviations

OFR: Ovarian follicular reserve
AMH: Anti Mullerian Hormone
NMRI: Naval Medical Research Institute
CTL: Control
FV: *Foeniculum vulgare*
LU: *Linum usitatissimum*
PND: Post-natal Day
TGF-β: Transforming growth factor-β
PD: Pregnancy Day
rpm: Revolutions per minute
H&E: Hematoxylin and eosin
IHC: Immunohistochemistry
PBS: Phosphate Buffered Saline
DAB: 3, 3-Diaminobenzidine
PCR: Polymerase chain reaction
cDNA: Complementary DNA
qRT-PCR: Quantitative Reverse Transcription PCR
FSH: Follicle stimulating hormone
PDGF: Platelet-derived growth factor
bFGF: Basic fibroblast growth factor
LIF: Leukemia inhibitory factor
KITL: Kit-ligand
BMP4: Bone-morphogen-protein 4
SDF1: Stromal-Derived-Factor1
